# Immunoglobulin light chain allelic inclusion in systemic lupus erythematosus

**DOI:** 10.1002/eji.201545599

**Published:** 2015-06-24

**Authors:** Louise D. Fraser, Yuan Zhao, Pamela M. K. Lutalo, David P. D'Cruz, John Cason, Joselli S. Silva, Deborah K. Dunn‐Walters, Saba Nayar, Andrew P. Cope, Jo Spencer

**Affiliations:** ^1^ Programme in Infection and ImmunobiologyKing's College LondonLondonUK; ^2^ Louise Coote Lupus Unit Guy's and St Thomas’ NHS TrustLondonUK; ^3^ Academic Department of RheumatologyKing's College LondonLondonUK

**Keywords:** Allelic inclusion, Immunoglobulin, Igκ, Igλ, Light chain, Systemic lupus erythematosus

## Abstract

The principles of allelic exclusion state that each B cell expresses a single light and heavy chain pair. Here, we show that B cells with both kappa and lambda light chains (Igκ and Igλ) are enriched in some patients with the systemic autoimmune disease systemic lupus erythematosus (SLE), but not in the systemic autoimmune disease control granulomatosis with polyangiitis. Detection of dual Igκ and Igλ expression by flow cytometry could not be abolished by acid washing or by DNAse treatment to remove any bound polyclonal antibody or complexes, and was retained after two days in culture. Both surface and intracytoplasmic dual light chain expression was evident by flow cytometry and confocal microscopy. We observed reduced frequency of rearrangements of the kappa‐deleting element (KDE) in SLE and an inverse correlation between the frequency of KDE rearrangement and the frequency of dual light chain expressing B cells. We propose that dual expression of Igκ and Igλ by a single B cell may occur in some patients with SLE when this may be a consequence of reduced activity of the KDE.

## Introduction

Functional B‐cell receptors are generated by a hierarchical series of Ig gene rearrangement events initiated during B‐cell development in the bone marrow 1, 2, 3. Following successful IgH rearrangement the IgL genes rearrange starting with the kappa IgL (Igκ) locus. If one allele of Igκ rearranges in the correct genetic reading frame and can successfully pair with the productively rearranged heavy chain, Igκ protein is expressed with IgH on the cell surface. If rearrangement at both Igκ alleles is unsuccessful, rearrangement of the IgL lambda (Igλ) locus is initiated. If a B‐cell achieves successful rearrangement of IgH and Igκ or Igλ, the B cell is then exposed to cellular and molecular processes that regulate central tolerance in the B‐cell system. This can be achieved by further gene rearrangements, either de novo or by editing existing light chain rearrangements 4, 5, 6, 7, 8.

Most B cells released into the circulation from the bone marrow contain multiple Ig light chain gene rearrangements as a consequence of possible sequential rearrangements of the Igκ alleles, and in some cells an additional one or two rearrangements at the Igλ locus. Moreover, approximately 30% of B cells with functional Igλ rearrangements also have potentially functional Igκ rearrangements that are in the correct genetic reading frame with no stop codons 9. Therefore the inactivation of unwanted rearranged Igκ, in particular those that are potentially functional and could be expressed, is an essential mechanism for maintenance of allelic and isotypic exclusion at the light chain loci in healthy individuals. Inactivation or deletion of unwanted Igκ rearrangements is achieved by rearrangement of the kappa deleting element (KDE), located downstream of the IgκC. KDE rearranges by RAG protein‐mediated recombination to either an intronic recombination signal sequence (iRSS) between the most 3’ IgκJ segment and IgκC, or to an upstream unrearranged IgκV segment 10, 11, 12, 13. If KDE rearranges to an upstream IgκV the unwanted VJ rearrangement is deleted, but in most cases KDE rearranges to the iRSS and the unwanted VJ rearrangement remains intact. In either case, KDE rearrangement deletes IgκC and the intronic enhancer 10, 11, 12, 13.

The mechanisms described above maintain allelic exclusion, so that despite the potential presence of multiple IgL rearrangements in a B cell, only a single IgH and IgL pair are expressed. Models of allelic inclusion in mouse B cells have been described where two light chains are expressed simultaneously 14. This may be associated with an autoimmune phenotype, though not necessarily with autoreactivity 15, 16, 17, 18. Failure of KDE rearrangement can associate with dual expression of Igk and Igλ by human B cells; a feature that has been identified in a small percentage of cells in healthy adults 19, 20, 21, 22

We have studied light chain expression by B cells from patients with systemic lupus erythematosus (SLE) and healthy controls. SLE is a chronic autoimmune disease characterised by polyclonal B‐cell hyperactivity and the production of pathogenic autoantibodies targeting self‐DNA and nucleoproteins 23, 24, 25, 26. We demonstrate that B cells from patients with SLE may express Igκ and Igλ simultaneously, that this is rare in healthy controls or patients with another systemic autoimmune disease granulomatosis with polyangiitis (GPA), and we provide evidence suggesting that light chain allelic inclusion can be a consequence of deficient KDE rearrangement in SLE.

## Results

### Immunoglobulin light chain allelic inclusion by B cells

**Figure 1:**
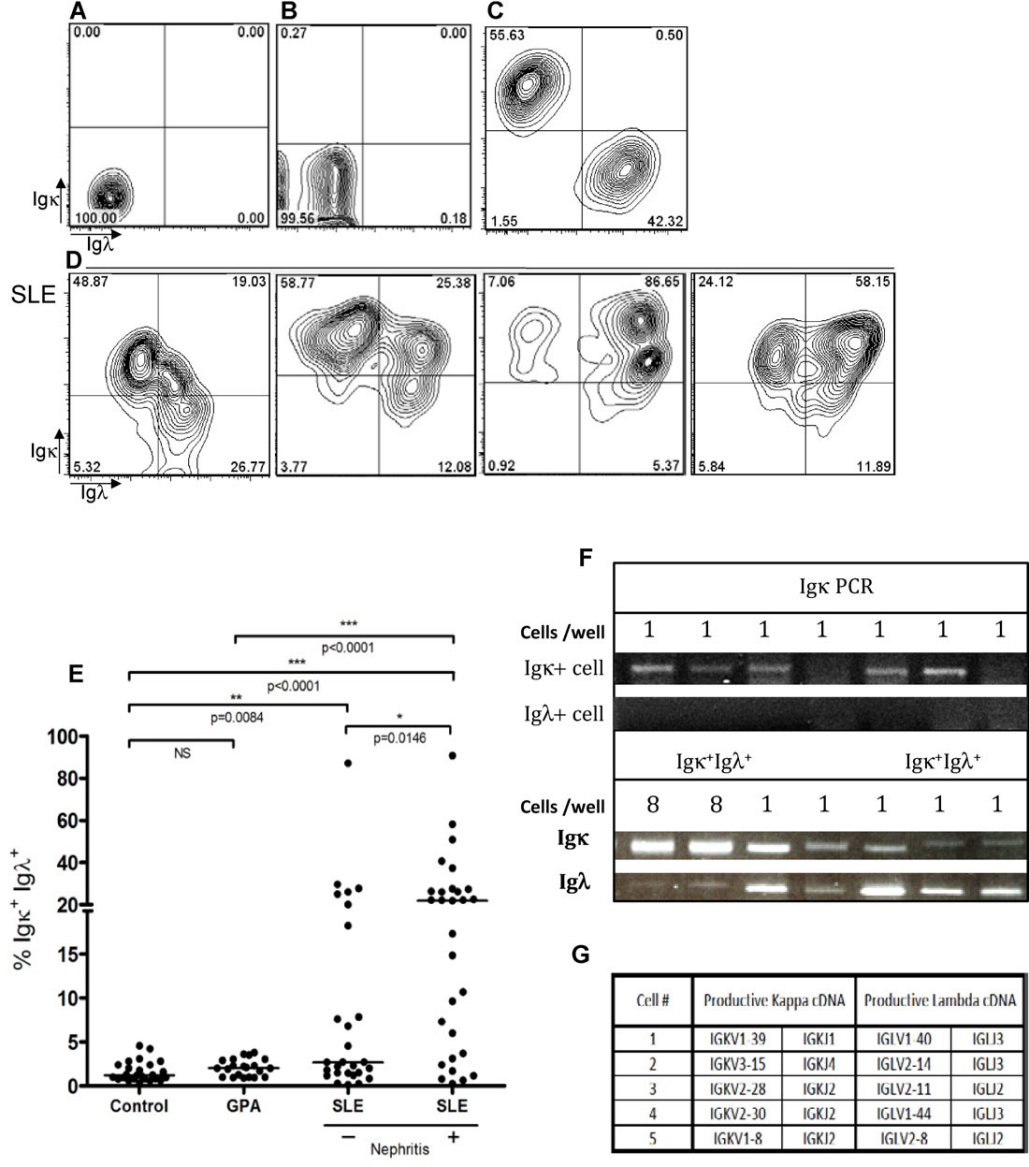
Expression of both Igκ and Igλ light chain by CD19^+^ B cells in SLE. CD19^+^ cells from healthy donors (*n* = 26) and SLE patients (*n* = 56) were analysed for cell surface expression of Igκ and Igλ by flow cytometry. (A and B) Representative contour plots of isotype controls for cell surface expression of Igκ and Igλ by CD19^+^ cells from a healthy donor and a patient with SLE, respectively. (C) Representative contour plot of cell surface expression of Igκ and Igλ by CD19^+^ cells in healthy donors showing clear separation of B cells expressing Igκ and Igλ. (D) Four representative contour plots of highly variable dual Igκ and Igλ expression by CD19^+^ B cells in patients with SLE. (E) Frequencies of viable CD19^+^ B cells expressing both Igκ and Igλ light chains in healthy donors (*n* = 26) SLE patients (*n* = 56) and patients with GPA (*n* = 23). Each dot shows an individual patient, bars represent averages. (F) Examples of PCR amplification of Igκ gene rearrangements from six consecutive single B cells expressing Igκ and failure to amplify Igκ gene rearrangements from six consecutive single B cells expressing Igλ from healthy donors.   In contrast, samples of eight cells pooled and five single cells from patient 43 sorted for dual Igκ and Igλ light chain expression gave products for both Igκ and Igλ. These were not originally consecutive wells; the PCR products were re run in adjacent lanes to compare band sizes. Representative gels of three independent experiments are shown. We confirmed that the bands from these five single cells were indeed Igκ and Igλ gene rearrangments by sequencing. V and J pairings for these rearrangments are illustrated in G. Statistical analyses were performed by Mann–Whitney tests assuming nonparametric distributions *p < 0.05; **p < 0.005; ***p < 0.0001; **** = *p* < 0.00001.

Peripheral blood B cells from patients with SLE, patients with GPA and healthy controls were evaluated for their expression of Igκ and Igλ light chains by flow cytometry. While the vast majority of B cells from healthy donors and patients with GPA expressed either Igκ or Igλ but not both (Fig. 1 A–C), approximately 54% patients with SLE had populations of CD19^+^ B cells that stained positive for both Igκ and Igλ light chains (Fig. 1D). Where patients had frequencies of Igκ and Igλ dual expressing B cells above the range observed in normal blood or GPA, the average value was approximately 28%, with a maximum value of over 90%. Dual Igκ and Igλ expressing B cells were particularly enriched in patients with lupus nephritis (Fig. 1E). Allelic inclusion was not associated with autoantibody titres, or disease activity in SLE (Supporting Information Fig. 1).

Expression of light chains by single cells was investigated by PCR. B cells from healthy individuals were sorted for expression of either Igκ or Igλ, and cDNA from each cell was tested in PCR reactions for amplification of Igκ or Igλ rearrangements. The PCR efficiency for amplification of light chain genes that agreed with surface light chain expression was approximately 41%. No examples of wells with both Igκ^+^ and Igλ^+^ PCR amplicons, or amplification of the ‘wrong’ light chain were obtained from 288 single Igκ^+^ or Igλ^+^ B cells from healthy donors. An example of amplicons from seven consecutive wells following amplification of Igκ from Igκ or Igλ expressing B cells from a healthy donor is illustrated in Fig. 1F. SLE B cells expressing both Igκ^+^ and Igλ^+^ were placed at 1 cell per well in 384 wells of 96‐well PCR plates. Of these, 14 wells gave PCR amplicons of both Igκ and Igλ. (Fig. 1F), 43 gave amplicons for Igκ only and 61 gave amplicons for Igλ only. The PCR amplicons of Igκ and Igλ from each of five single cells illustrated in Fig. 1F were sequenced and were confirmed to be productively rearranged Igκ and Igλ rearrangements (Fig. 1G). Experimentally, these were not adjacent reactions. PCR products were run alongside positive controls with eight dual positive cells per well to allow direct comparison of band sizes. Although the PCR efficiency for dual Igκ^+^ or Igλ^+^ B cells was low, the frequency of detection of amplicons for both Igκ and Igλ from a single cell was significantly greater in dual Igκ and Igλ^+^ positive cells from SLE patients than in the single Igκ^+^ or Igλ^+^ positive cells in healthy donors by χ2 analysis (*p* = 0.001). The reason for PCR inefficiency for dual Igκ^+^, Igλ^+^ positive cells is not clear.

**Figure 2:**
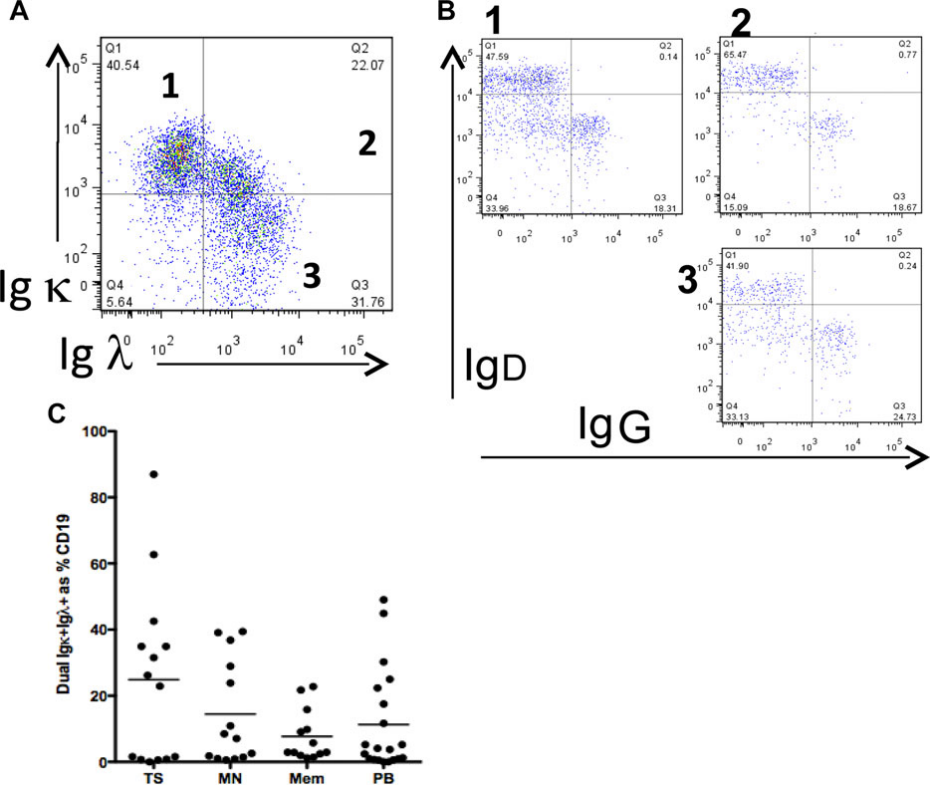
(A) PBMCs from SLE patient 119 were stained for CD19, IgD, IgG, Igκ, and Igλ and CD19^+^ B cells expressing single Igκ ( quadrant 1), single Igλ (quadrant 3), or both Igκ and Igλ ( quadrant 2) were identified by flow cytometry. (B) Cells in quadrants 1, 2, and 3 from A were then regated to identify cells with surface IgD and IgG. (A, B) One representative plot out of two independent experiments is shown. (C) Quantification of flow cytometric analysis of the frequency of Igκ and Igλ light chain expression in different B‐cell subsets in SLE; transitional (TS) B cells (CD19^+^ IgD^+^ CD27^−^ CD10^hi^), mature naive (MN) B cells (CD19^+^ IgD^+^ CD27^−^ CD10^lo^), memory (Mem) B cells (CD19^+^ IgD^−^ CD27^+^), and plasmablasts (PB) (CD19^+^ CD38^hi^ CD27^hi^). Each dot shows an individual patient, bars show averages.

If the presence of both light chains were a consequence of binding of polyclonal IgG to the B‐cell surface, then the heavy chain of IgG for example would be evident on all double‐positive cells, which it was not (Fig. 2A and B). To investigate whether dual light chain expression was a feature of a particular B‐cell compartment, we examined the profile of light chain expression on transitional (TS) B cells (CD19^+^ IgD^+^ CD27^−^ CD10^hi^), mature naive (MN) B cells (CD19^+^ IgD^+^ CD27^−^ CD10^−^), memory (Mem) B cells (CD19^+^ IgD^−^ CD27^+^ CD10^−^), and plasmablasts (PB) (CD19^+^ CD38^hi^ CD27^hi^) by flow cytometry. Wherever expression of both Igκ and Igλ light chains by CD19^+^ B cells was present, it was observed in all B cell subsets studied (Fig. 2C).

**Figure 3:**
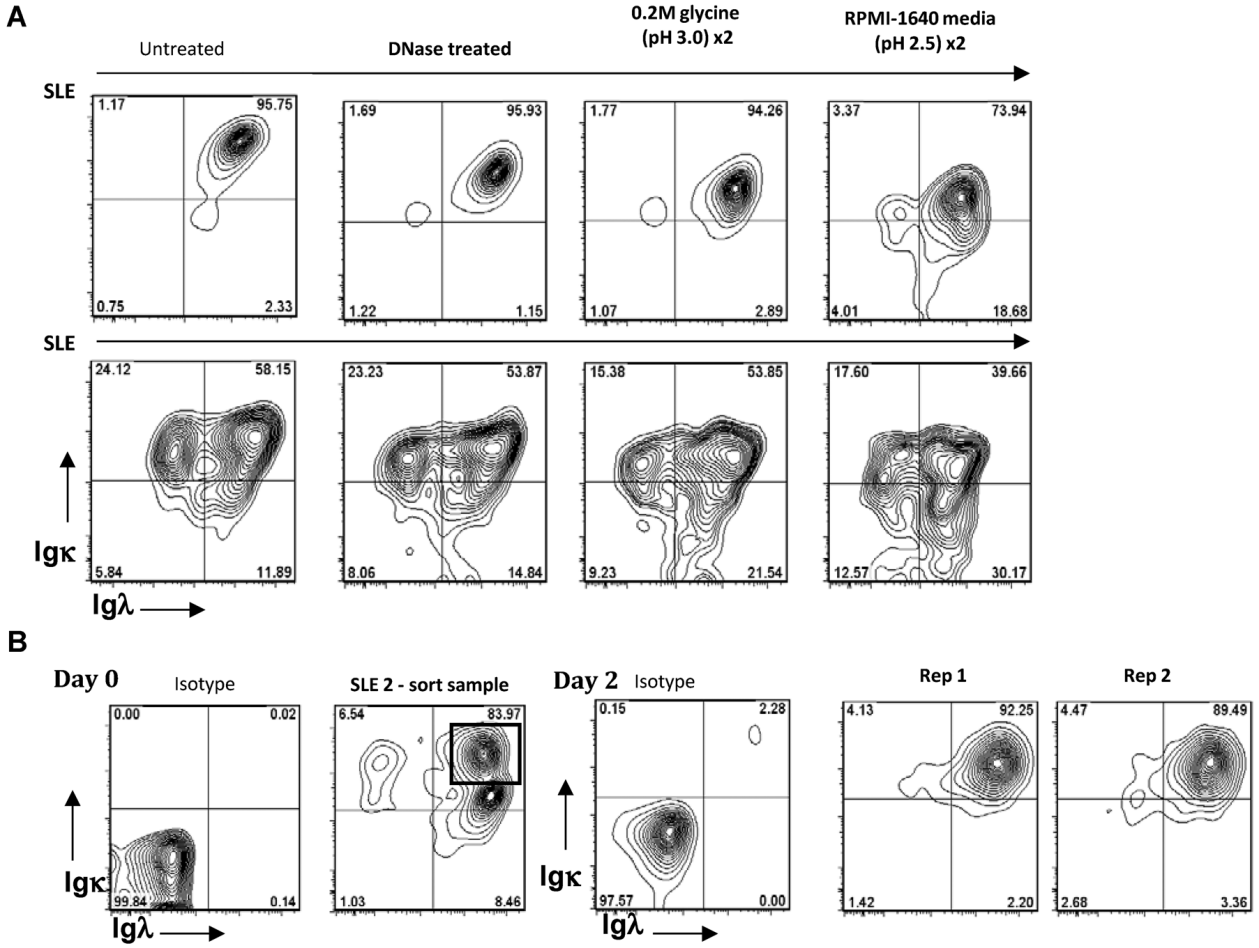
Cell surface expression of Igκ and Igλ light chains following DNAse treatment, acid washes, and cell culture. (A) Flow cytometry analysis of light chain expression by CD19^+^ SLE B cells following incubation with DNase, or two acid wash steps using two different acidic wash buffers; 0.2 M glycine (pH3.0) and RPMI‐1640 media wash (pH2.5) prior to immunostaining and flow cytometric analysis. Representative contour plots from three independent experiments with three different donors are shown. (B) Immunoglobulin light chain cell surface expression on sorted Igκ^+^, Igλ^+^ CD19^+^ B cells from an SLE patient was determined by flow cytometry on day 0 or following coculture with allogeneic CD3^+^CD4^+^ T cells with PMA for 2 days. Isotype controls and two replicates (Rep1 and Rep2) out of two independent experiments with two different donors are shown.

We considered a range of possible explanations for the presence of both Igκ and Igλ on B cells, including binding of polyclonal Ig to surface receptors. To explore if binding of exogenous complexes of DNA and anti‐DNA antibodies could account for detection of both Igκ and Igλ light chains on B cells, PBMCs from SLE patients were incubated with DNase prior to immunostaining. Dual light chain cell surface expression was still evident (Fig. 3A). To further eliminate the possibility that the detection of both light chains on cell populations was a consequence of FcR‐mediated binding of serum immunoglobulin to the B‐cell surface, cells were washed twice in 0.2 M glycine (pH 3.0) or RPMI‐1640 media (pH 2.5) acidic wash buffers to elute such complexes prior to immunostaining and flow cytometric analysis. This too did not alter the flow cytometric profile (Fig. 3A).

To determine whether the expression of both Igκ and Igλ was stable, dual light chain‐expressing SLE B cells were isolated by FACS and cultured with allogeneic CD3^+^CD4^+^ T cells and PMA for two days. Cells remained positive for both light chains after the activating culture period (Fig. 3B).

The presence of Igκ and Igλ light chains on the cell surface was not a feature of dying cells and was not a feature of PBMCs other than the B‐cell subsets described (Supporting Information Fig. 2).

### Dual positive B cells had both Igκ and Igλ on the cell surface and in the cytoplasm

**Figure 4:**
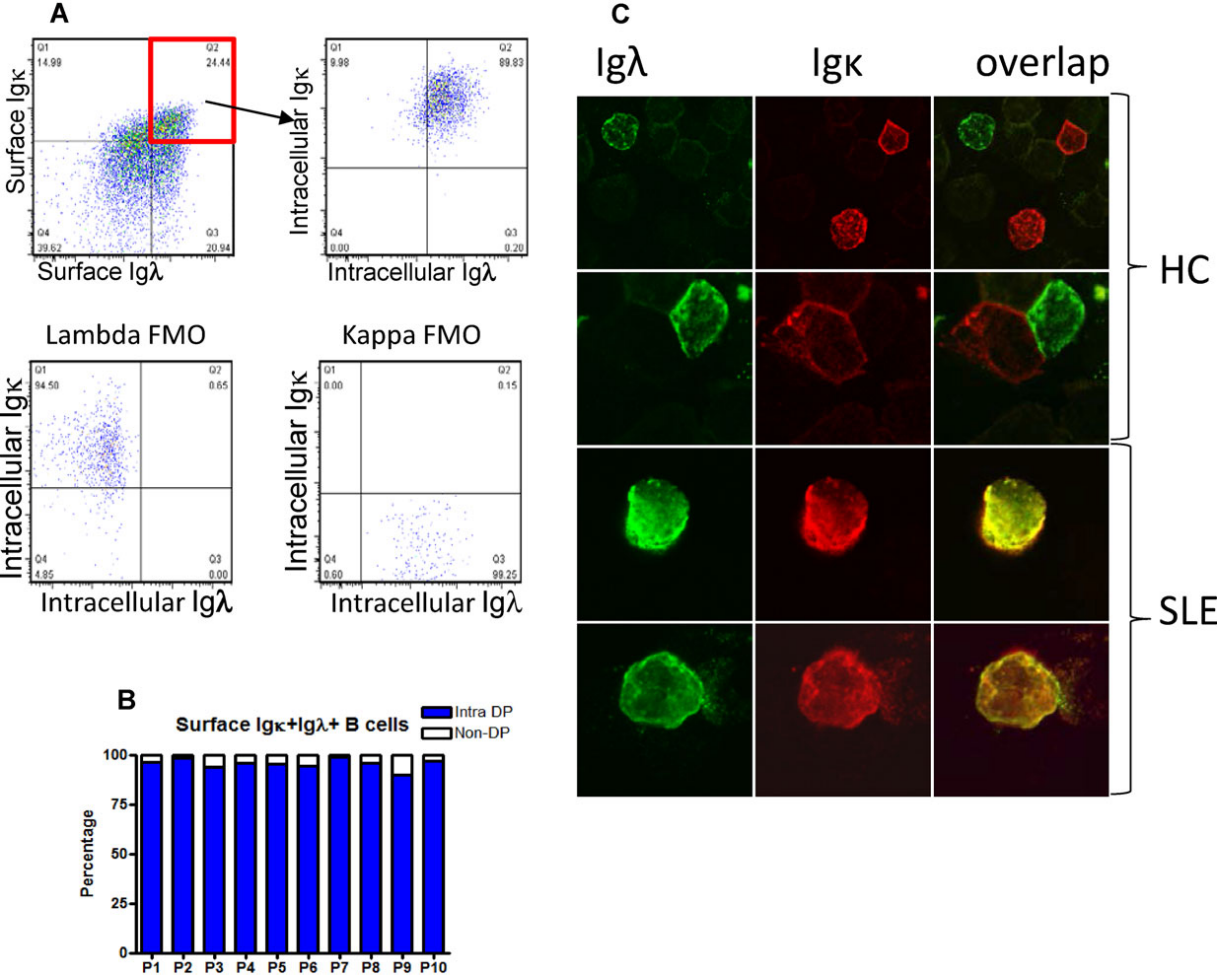
Dectection of surface and intracellular Igκ and Igλ light chains by CD19^+^ B cells in SLE. (A) B cells from a patient with SLE gated to identify B cells with surface Igκ and Igλ, remain Igκ^+^ and Igλ^+^ when reanalysed for cytoplasmic Igκ and Igλ. Fluorescence minus one (FMO) controls ae illustrated. One representative dot plot out of ten independent experiments is shown. (B) This was quantified and found to be consisent in ten patients previously seen to show dual light chain expression. Intra‐DP refers to the percentage of B cells that are double positive for surface Igκ and Igλ, that are then shown to be Igκ and Igλ following permeabilisation and use of antibodies to Igκ and Igλ conjugated to different fluorochromes. Bars show the percentage of intracellular double‐positive cells out of the total surface double‐positive B cells for ten patients analysed. (C) B cells from healthy controls and SLE patients were stained for Igκ and Igλ expression and analysed by confocal microscopy. Representative images from two independent experiments with four donors are shown. Original magnification ×60.

PBMCs from patients with SLE known to show allelic inclusion were stained to detect surface CD19, Igκ, and Igλ. Cells were then fixed and permeablised and stained for Igκ and Igλ with antibodies conjugated to different fluorochromes. The vast majority of cells that had both Igκ^+^ and Igλ^+^ on the cell surface also had intracytoplasmic Igκ and Igλ (Fig. 4A, B) by this method.

PBMCs from SLE patients with a high frequency of Igκ and Igλ^+^ cells by flow cytometry were spun onto glass slides using a cytocentrifuge. Cells were then permeablised and stained to identify Igκ and Igλ. Whereas healthy B cells were consistently either Igκ^+^ or Igλ^+^, lymphocytes with a superimposed distribution of Igκ and Igλ on the cell surface and in the cytoplasm could consistently be observed in cases of SLE that expressed both light chains by flow cytometry (Fig. 4C).

### Lower frequency of KDE rearrangement in SLE inversely correlates with dual light chain expression

**Figure 5:**
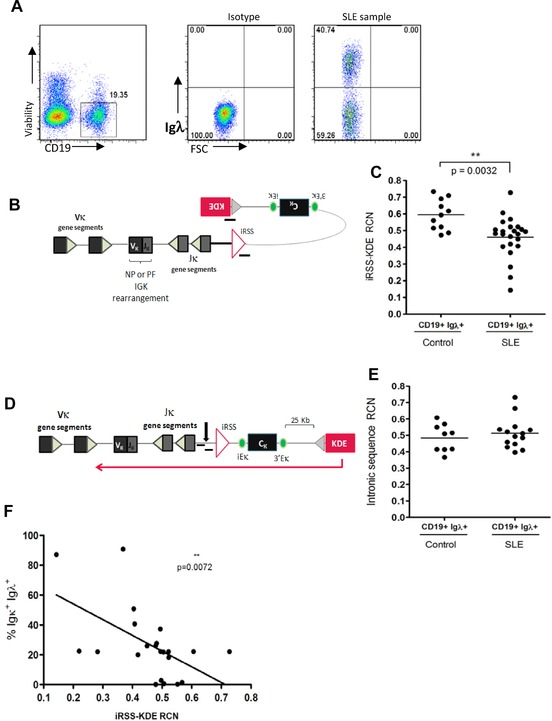
Relationship between frequency of KDE rearrangement to the iRSS and dual light chain expression. (A) Sorting strategy to avoid biases resulting from gating. Cells expressing Igλ were sorted, irrespective of whether they also expressed Igκ prior to isolation of genomic DNA. One representative dot plot out of 34 independent experiments is shown. (B) Representation of qRT‐PCR amplification of rearranged KDE/iRSS junctions. (C) Relative copy numbers (RCN) of iRSS‐KDE rearrangments in gDNA isolated from FACS sorted CD19^+^ Igλ ^+^ B cells from healthy controls (*n* = 11) and SLE patients (*n* = 23) were determined by qRT‐PCR as shown in (B). RNaseP was used as a reference gene and data was adjusted in each assay so that cell line NALM6 had a value of 2. Each dot shows an individual patient, bars show means. (D) Representation of qRT‐PCR amplification of intronic sequence between the iRSS and IgKJ. (E) Relative quantification by qRT‐PCR of an intronic region of the Igκ light chain, located 5’ to the iRSS site, in gDNA isolated from CD19^+^ Igλ ^+^ B cells from healthy controls (*n* = 9) and SLE patients (*n* = 14) standardised to RNaseP. Each dot shows an individual patient, bars show means. (F) RCN of iRSS‐KDE rearrangements standardised to RNaseP is inversely correlated with the frequency of dual light chain expressing cells. Each dot shows an individual patient, and regression line is illustrated. Statistical analysis was performed by Mann–Whitney tests assuming nonequal variance (C, E) or Spearman's test for correlation (F) where **p < 0.01.

Rearrangement of the KDE normally functionally inactivates rearrangements of *Igκ* that are not used by the cell, by deleting the constant region and the intronic enhancer. Since productively rearranged but nonexpressed Igκ segments are carried by approximately 30% of Igλ expressing B cells 9 we considered the possibility that failure of KDE rearrangement, that has also been described previously in SLE 29, could permit aberrant expression of both light chains simultaneously. To ask whether the expression of both Igκ and Igλ could be the result of a reduced frequency of rearrangement of the KDE in the patients studied, iRSS‐KDE rearrangements were quantified in the genomic DNA isolated from FACS‐sorted populations of CD19^+^Igλ^+^ B cells from SLE patients and healthy donors by quantitative real‐time PCR (qRT‐PCR) (Fig. 5A–C). The frequency of iRSS‐KDE rearrangements in Igλ—expressing B cells in SLE was significantly lower than observed in healthy donors (Fig. 5C).

To ask whether the reduced frequency of rearrangement of the KDE to the iRSS observed in SLE compared to healthy donors was due to more frequent KDE rearrangement to IgκV segments rather than the iRSS, qRT‐PCR primers were designed to target the IgκJ‐IgκC intron, 5’ of the iRSS in the same genomic DNA samples from which iRSS‐KDE rearrangement frequency had been determined (Fig. 5D). The frequency of KDE rearrangements to this intronic sequence was equivalent in CD19^+^ Igλ^+^ B cells from healthy donors and patients with SLE (Fig. 5E), indicating that there was no difference in the frequency of KDE rearrangement to *IgκV* segments in healthy donors and SLE. This observation also provided a control to demonstrate that the reduced frequency of KDE rearrangements observed in sorted B cells from patients with SLE in Fig. 5C was not a consequence of inclusion of B cells expressing Igκ only in the sorted populations of cells since the same DNA samples were used for both sets of experiments.

To test if there was any association between reduced frequency of rearrangement of KDE to iRSS in a population of cells and allelic inclusion in SLE, the frequencies of B cells expressing both Igκ and Igλ light chains and the relative frequency of rearrangement of KDE in Igλ expressing B cells in that case were plotted as an XY graph. We identified an inverse correlation between the frequencies of B cells expressing both Igκ and Igλ light chains and the frequency of rearrangement of KDE in Igλ expressing B cells, implying that expression of both light chains is associated with lower frequency of rearrangement of KDE to iRSS (Fig. 5F).

**Figure 6:**
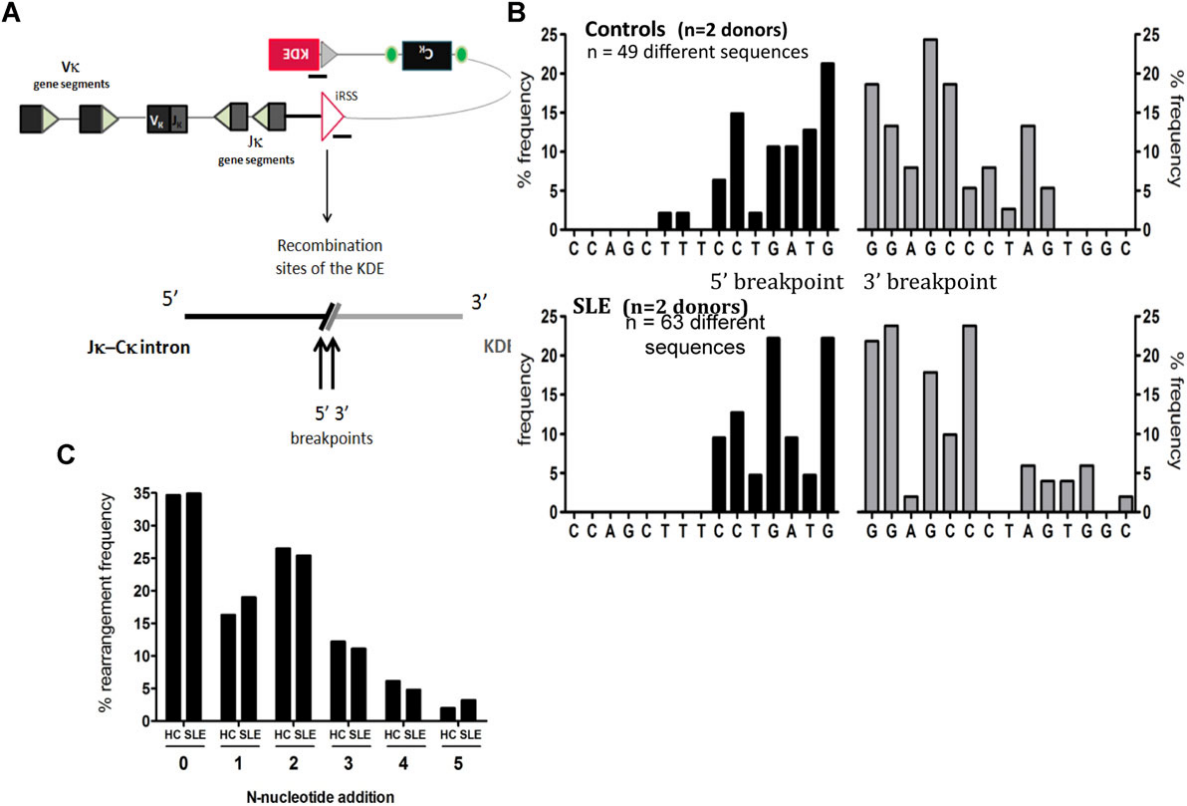
Comparison of iRSS‐KDE junctions and sequences flanking the junctions in B cells from healthy donors and patients with SLE. (A) KDE rearrangements to the iRSS were PCR amplified from gDNA isolated from CD19^+^Igλ^+^ B cells from healthy controls (*n* = 2) and SLE patients (*n* = 2). PCR products were cloned, sequenced, and aligned to germline sequences. (B) Bars show the frequency of sequences that terminate at specific breakpoints after iRSS‐KDE rearrangement. (C) Frequency of N‐nucleotide additions to the iRSS‐KDE junction in healthy donors (*n* = 2) and SLE patients (*n* = 2) was determined as shown in (A). Bars show percentage of sequences with each number of nucleotide additions to the junction.

It was considered possible that reduced KDE rearrangement to the iRSS but not to IgκV in SLE might be due to differences in the sequences around the recombination sites or the properties of the KDE rearrangement itself. Therefore, the junctions and flanking sequences of rearrangements between the KDE and iRSS were sequenced following the cloning of PCR‐amplified iRSS to KDE rearrangements from genomic DNA extracted from CD19^+^ Igλ^+^ B cells from two healthy controls and two SLE patients with B cells expressing both light chains. The experiment outline is illustrated in Fig. 6A. A total of 49 different sequences from healthy controls and 63 from SLE patients were aligned with the germline sequence of the intron between IgκJ and IgκC and germline KDE sequence to identify the breakpoints and to quantify nucleotide additions. No differences were observed between healthy B cells and SLE B cells at the intronic RSS and KDE breakpoint or the sequences flanking them (Fig. 6B). No differences in the frequency of N‐nucleotide additions at the junctions were observed (Fig. 6C).

## Discussion

We report that B cells may express both Igκ and Igλ light chains in some cases of SLE. Light chain isotypic inclusion, that can affect more than 90% of B cells, was more common in B cells from SLE patients with more severe disease (as manifest by lupus nephritis) but was independent of disease activity. In contrast, dual light chain expression in healthy donors is a relatively rare event that has been observed previously in healthy subjects with frequencies of 0.2–2% of B cells 19, 20, 21, 22, consistent with our observations. Light chain isotypic inclusion is not a feature of all autoimmune diseases, since it was not seen in blood of patients with GPA.

B cells expressing more than one unique light chain in mice have been shown to exhibit autoreactivity, and to be present with relatively high frequency in autoimmune prone mice 14, 15, 16. In mouse models, their frequency also increases with age and autoimmune disease severity. They are present within both plasmablast and memory B‐cell populations 14, 15, 16, and associated with receptor editing where one light chain rearrangement may replace another with unwanted specificity 15, 17, 18. Our data suggest that these observations in mouse models of SLE are highly relevant to human disease and that expression of both light chains simultaneously may contribute significantly to disease pathogenesis in the subset of patients affected.

Surprisingly, IgL allelic inclusion associated with autoimmunity or inflammation has not been described in humans previously despite numerous studies of B cells in SLE. Light chain usage has been studied at a single cell level by RT‐PCR of RNA encoding IgH and IgL genes 27. Our analysis suggests that detection of both light chains by single cell RT‐PCR is relatively inefficient and this could result in failure to detect both expression of both light chains by PCR alone In addition, if PCR amplicons for two light chains are obtained from a single B cell in a well, the logical conclusion might be to assume that two cells were placed in the well, and to discard this data. The data obtained by analysis of DNA of single cells is difficult to interpret. We now know that cells can carry multiple light chain gene rearrangements even in healthy B cells 9 and that these are clearly not all used, so analysis of IgL rearrangements in B cells by PCR of DNA in general may be difficult to interpret.

Dual light chain expression could represent failure in efficient receptor editing. The KDE may fail to inactivate the unwanted rearrangement that has been selected against, so that the new and the old rearrangements are both retained. Receptor editing is known to be an important regulator of the maturing B‐cell repertoire that prevents the survival and maturation of self‐reactive cells 17, 18 and allelic inclusion has been associated with receptor editing 15. Alternatively the phenotype could represent a mid‐way stage in receptor revision if expression of one light chain is replaced by expression of another in mature B cells, again by failure of the KDE to inactivate the unwanted rearrangement 28. If light chain revision occurs, the exchange of protein on the cell surface may not mirror the tempo of RNA change that would be a balance between RNA production at both light chain loci and extinguishing of activity of the original rearrangement by KDE rearrangement. This latter possibility could potentially explain the inefficiency of the single cell PCR. The processes that initiate and regulate KDE activity and specifically direct the KDE to the iRSS are not known and it is difficult therefore to speculate as to why this may be defective in SLE.

The chronic expression of two light chains and the assembly of IgH with either light chain on the cell surface could increase the antigen‐binding potential of B cells as well as the range of cognate peptides presented to CD4 T cells as a consequence of a greater diversity of internalized antigens. It is possible that a single B‐cell receptor could have different specificities on the two arms so that complex antigenic repertoires might be generated 16. Dual light chain expressing B cells could differ in their ability to initiate and escalate pathogenic immune responses. Expression of both light chains was not associated with a particular B‐cell subset in SLE, but was apparent through the entire spectrum of B‐cell differentiation from transitional B cells through to plasmablasts, and thus the aberrant receptors they express could contribute to B‐cell responsiveness and function at all points in B‐cell development.

It is interesting that allelic inclusion is associated most closely with lupus nephritis. It is possible that some of the patients with SLE classified as not having nephritis but exhibiting high frequencies of dual Igκ^−^ and Igλ^−^expressing B cells may develop the disorder in the future. These patients will be followed up to test if presence of dual Igκ‐ and Igλ^−^ expressing B cells could be a prognostic biomarker of more severe disease.

In summary, we have demonstrated that B cells in some cases of SLE can express both Igκ and Igλ simultaneously, and that this is associated with lower frequency of rearrangement of the KDE described here and in a previous study 29. Expression of multiple B‐cell receptor specificities by a single cell could enhance the potential responsiveness of B cells to antigen and the spectrum of peptides they can present.

## Materials and methods

### Patients and peripheral blood samples

Approval for collection of blood samples from SLE patients and controls for this study was given by the KCL Infectious Diseases BioBank working under the authority of the Southampton and South West Hampshire Research Ethics Committee (REC Approval: REC09/H0504/39—‐The Control of Inflammation in Immunity and Autoimmunity; IDB ref JS‐1b). All SLE patients fulfilled the American College of Rheumatology classification criteria for SLE, with at least four of the 11 criteria present, serially, or simultaneously 30, 31. They were all female with average age of 38 years, range 17–73. Information on organ involvement, auto‐antibody titres and disease duration are in Supporting Information Table 1. GPA patients studied had an average of 54 years (range 27–71) and clinical details are in Supporting Information Table 2.

### Antibodies and flow cytometry and confocal microscopy

PBMCs were isolated from blood or buffy coats by Ficoll–Hypaque centrifugation for 25 min at 400 g. PBMCs were stained with CD19^−^PerCP‐Cy5.5, kappa light chain allophycocyanin‐H7 (BD Biosciences Pharmingen) and lambda light chain‐Pacific Blue (Biolegend) and LIVE/DEAD Fixable Aqua (Life Technologies) for 15 min at 4°C before analysis on the FACSCanto (BD Biosciences Pharmingen) for cell surface expression of CD19, Igκ, and Igλ. For intracytopasmic staining of light chains, cells were initially stained as described above, and then permeabilised using Fixation/Permeabilization buffer (eBiosciences) before staining to detect cytoplasmic Igκ with kappa light chain—AF647 (BioLegend) and Igλ with lambda light chain—AF488 (BioLegend).

Flow cytometric analysis of B‐cell subsets involved additional staining PBMCs with IgD^−^PE and CD27^−^PE‐Cy7 (BD Biosciences Pharmingen), CD10^−^allophycocyanin (Biolegend), and CD38^−^PE (eBioscience) where TS B cells were defined as CD19^+^IgD^+^CD27^−^CD10^hi^, MN B cells as CD19^+^IgD^+^CD27^−^CD10^−^, memory (Mem) B cells as CD19^+^IgD^−^CD27^+^ and plasmablasts (PB) as CD19^+^CD27^hi^CD28^hi^ 32. Data were analysed by FACS Diva. Cells were sorted on the FACSAria machine (BD Biosciences Pharmingen).

For confocal microscopy, cells were spun onto microscope slides by cytocentrifuge and fixed and permeabilised with acetone. They were then stained for Igκ with kappa light chain‐–AF647 (BioLegend) and Igλ with lambda light chain—AF488 (BioLegend). Cells were visualized using a Leica DMIRE2 confocal microscope.

### Removal of immune complexes and cytophilic binding of serum Ig

PBMCs were DNase treated (0.1 mg/mL for 30 min at room temperature) before washing and staining for CD19, Igκ, and Igλ. Acidic wash buffers were made by adjusting a 0.2 M glycine solution to pH3, and RPMI‐1640 medium pH 2.5 with acetic acid. Total PBMCs were washed twice for 5 min at 1200 rpm before staining for CD19, Igκ, and Igλ.

### Dual expression of Igκ and Igλ after B‐cell activation in culture

CD19^+^ B cells from an SLE patient with both Igκ and Igλ light chains on their surface were FACS‐sorted and cultured at a 1:1 ratio with sorted allogeneic CD3^+^CD4^+^ T cells and PMA (50 ng/mL). Cells were harvested on day 2 and restained for cell surface expression of CD19, Igκ, and Igλ.

### PCR amplification of rearranged Igκ and Igλ transcripts from cDNA isolated from single cells

CD19^+^ B cells expressing both Igκ and Igλ simultaneously were FACS sorted into 96‐well plates containing 18 μL of sort lysis reverse transcription buffer per well. Following sorting, 2 μL 1:8 SuperScript III (Invitrogen) was added to each well prior to reverse transcription.

Primer sequences are in Supporting Information Table 2. Semi‐nested PCRs were used to detect and amplify V‐C transcripts for both Igκ and Igλ light chain gene rearrangements in the cDNA of sorted single cells. First round PCR reactions were performed in a 20 μL volume; 4 μL 5X GC buffer (New England Biolabs), 2 μL 1:20 Phusion DNA polymerase (0.2 U/mL) (New England Biolabs), 0.2 μL dNTP mix (20 mM each, Promega), 1 μL Vk1‐6 or Vl1‐8(5’) PCR1 primer mix (835 nM or 1000 nM, respectively), VkC or VlC (3’) PCR1 primer mix (5 mM each), 1 μL 2.0 M MgCl_2_, 8.8 μL PCR‐grade H_2_0 and 3 μL of cDNA. Second round PCR reaction was also performed in a 20 μL volume; 4 μL 5X GC buffer, 2 μL 1:20 Phusion DNA polymerase (0.2 U/mL), 0.2 μL dNTP mix (20 mM each, Promega), 1 μL Vk1‐6 or Vl1‐8(5’) PCR2 primer mix (835 nM or 1000 nM, respectively), VkC or VlC (3’) PCR2 primer mix (5 mM each), 10.8 μL PCR‐graded H_2_0 and 2 μL PCR1 product.

PCR cycle conditions for Vk‐Ck were as follows; PCR1: 98°C for 30 s, followed by 50 cycles of 98°C for 10 s, 58°C for 15 s, and 72°C for 40 s. Final extension at 72°C for 10 min. PCR2: 98°C for 30 s, followed by 20 cycles of 98°C for 10 s, 64°C for 15 s, and 72°C for 15 s. Final extension at 72°C for 10 min. cycle conditions for Vl‐Cl: PCR1: 98°C for 30 s, followed by 50 cycles of 98°C for 10 s, 58°C for 15 s, and 72°C for 30 s. Final extension at 72°C for 10 min. PCR2: 98°C for 30 s, followed by 20 cycles of 98°C for 10 s, 62°C for 15 s, and 72°C for 15 s. Final extension at 72°C for 10 min.

### Quantitative RT‐PCR

Quantitative real‐time PCR for the amplification of rearranged iRSS‐KDE junctions were designed by Applied Biosystems using a Taqman Gene Expression Assay. iRSS‐KDE rearrangements were amplified as described by 29. All qRT‐PCRs were performed in multiplex, with quantification of iRSS‐KDE rearrangements standardised to the Taqman Copy Number Reference Assay, RNaseP, detected with a VIC‐labelled probe (Applied Biosystems). Relative copy numbers were standardised to the cell line NALM6 that has two rearrangements of KDE.

Quantitative real‐time PCR for the amplification of an intronic region 5’ to the Jk‐Ck intronic RSS were also produced by Applied Biosystems using a similar Taqman Gene Expression Assay. This region was amplified with the following primers 5’‐GGGTCTGATGGCCAGTATTGAC‐3’ and 5’‐CCAATACAATATTTGGCAAG‐3’ and were detected with a FAM‐labelled 5’‐AAAGATTGGTAAATGAGGGCATTTA‐3’ hydrolysis probe. This assay was also performed in multiplex qRT‐PCR reaction with the RNaseP reference assay.

### Amplification and sequencing of rearranged Igκ genes and iRSS‐KDE rearrangements

DNA was isolated from populations of FACS‐sorted CD19^+^ B cells using the DNeasy Blood & Tissue Kit (Qiagen). Primer sequences are shown in Supporting Information Table 2 25. First PCR amplification of IgκV‐IgκJ or iRSS‐KDE rearrangements was performed under the following cycle conditions: one cycle of 95°C for 7 min, 56°C for 1 min, and 72°C for 1 min 30 s. Followed by 30 cycles of 94°C for 1 min, 56°C for 30 s, and 72°C for 1 min 30 s. A final extension of 5 min at 72°C was performed. Five microliters of each first round PCR product was used in internal amplifications performed under the following conditions: one cycle of 95°C for 7 min, 65°C for 1 min, and 72°C for 1 min 30 s. Followed by 30 cycles of 94°C for 1 min, 65°C for 30 s, and 72°C for 1 min 30 s. A final extension of 5 min at 72°C was performed. PCR products were separated by gel electrophoresis and visualised with ethidium bromide. PCR products were cloned and sequenced as described previously 28, 33.

## Conflict of interest

The authors declare no commercial or financial conflict of interest.

**Abbreviations**
GPA: granulomatosis with polyangiitis
Igκ: immunoglobulin kappa light chain
Igλ: immunoglobulin lambda light chain
iRSS: intronic recombination signal sequence
KDE: kappa deleting element
MN: mature naive
SLE: systemic lupus erythematosus
TS: transitional

## Supporting information

As a service to our authors and readers, this journal provides supporting information supplied by the authors. Such materials are peer reviewed and may be re‐organized for online delivery, but are not copy‐edited or typeset. Technical support issues arising from supporting information (other than missing files) should be addressed to the authors.

Figure S1. Dual kappa and lambda light chain expression by SLE paEents posiEve and negaEve for the producEon of SLE–‐associated autoanEbodies.
Figure S2. Examples of gating to identify
Table S1. Demographic and clinical details of the SLE patients enrolled in this study
Table S2. Demographic and clinical details of the GPA patients enrolled in this study
Table S3 Primer sequences

Immunoglobulin light chain allelic inclusion in systemic lupus erythematosus
