# Supplementary material for: Immunoglobulin light chain allelic inclusion in systemic lupus erythematosus
Source: Eur J Immunol. 2015 Jun 24;45(8):2409–19. doi: 10.1002/eji.201545599 (PMC5102633; doi:10.1002/eji.201545599)
Supplement: Supplementary file 1 — Figure S1. Dual kappa and lambda light chain expression by SLE paEents posiEve and negaEve for the producEon of SLE–‐associated autoanEbodies. Figure S2. Examples of gating to identify Table S1. Demographic and clinical details of the SLE patients enrolled in this study Table S2. Demographic and clinical details of the GPA patients enrolled in this study Table S3 Primer sequences [file EJI-45-2409-s001.pdf]

Table S1. Demographic and clinical details of the SLE patients enrolled in this study

SLE

Study #	M/F	Age	Ethnicity	Disease duration (years)	+ Auto-Ab status	Nephritis	Arthritis	Medication	% of K+λ+ B cells
9	F	48	AfC	7	dsDNA, ANA, ENA, Sm, RNP	+	+	Pred., MTX, HCQ	0.26
11	F	42	Cauc	22	ANA, Ro, La, aPL	-	+	Pred., MTX, HCQ	0.13
14	F	57	Cauc	10	dsDNA, ANA, ENA, Ro	+	+	Pred., MMF	0.64
15	F	23	Cauc	8	dsDNA, ANA, ENA, Ro, RNP, aPL	-	+	HCQ	20.02
16	F	49	AfC	17	ANA, ENA, Ro, RNP	-	+	HCQ	18.23
17	F	45	Cauc	16	ANA, ENA, Ro, Sm, aPL	+	+	MMF, HCQ, Mepacrine	26.06
18	F	65	Cauc	8	ANA, ENA, Ro, Sm, RNP	-	+	no medication	1.45
22	F	73	Cauc	25	dsDNA, ANA, ENA, Ro	-	+	HCQ	27.67
23	F	45	Cauc	15	dsDNA, ANA, ENA, Ro, La, aPL	-	+	HCQ	2.71
24	F	45	Af	6	dsDNA, ANA, Ro, La	+	+	HCQ, Pred	37.32
25	F	40	Cauc	14	dsDNA, ANA, ENA, Ro, aPL	-	-	HCQ, Pred	87.06
26	F	53	Cauc	18	dsDNA, ANA, ENA, Ro, La	-	-	no medication	1.18
31	F	21	AfC	3	dsDNA, ANA, ENA, Ro	+	+	AZA, HCQ	58.15
39	F	40	Asian	16	dsDNA, ANA	+	+	AZA, HCQ, Pred	50.8
40	F	35	Orien	13	ANA, Ro, La	+	-	AZA, Pred	25.97
41	F	51	Cauc	9	dsDNA, ANA, Ro, Sm, RNP	-	-	MMF, HCQ, Pred	2.31
42	F	63	Cauc	20	ANA	-	+	AZA, Pred	0.82
43	F	46	Af	5	dsDNA, ANA, Ro, Sm, RNP	+	+	MMF, HCQ, Pred	90.73
44	F	54	Af	4	ANA, RNP	-	+	HCQ, Pred	4.53
45	F	40	Af	12	ANA, aPL	+	-	MMF, HCQ, Pred	14.85
46	F	23	Cauc	2	dsDNA, ANA, Ro, La	+	-	MMF, HCQ, Pred	1.18
47	F	59	Cauc	4	ANA	-	+	Leflunomide, HCQ	1.48
48	F	32	Cauc	3	ANA	-	+	HCQ, Pred	2.68
49	F	51	Cauc	4	ANA, Ro	-	-	none	0.31
56	F	58	Cauc	39	ANA, aPL	-	+	none	29.6

61	F	31	Af	5	dsDNA, ANA	-	+	Pred., HCQ, RTX 2008, 2009, 2011 2012, Thal	2.7
66	F	44	Cauc	18	dsDNA, ANA, ENA, RNP	+	+	MMF, HCQ, Pred	3.71
67	F	52	AfC	16	dsDNA, ANA, ENA, C1q	+	+	RTX 2012 HCQ, Pred	27.3
69	F	47	Af	20	dsDNA, ANA, ENA, Ro, Sm, RNP, C1q	+	+	CYC IV HCQ Pred	27.5
70	M	62	Cauc	20	ANA, ENA, Ro, La	-	+	HCQ, Pred	25
71	F	67	AfC	1	ANA, ENA, Sm, RNP	-	+	RTX 2012 HCQ, Pred	1.8
73	F	33	Af	9	ANA, ENA, Ro, Sm	-	+	HCQ, Pred	7.84
75	F	56	Cauc	12	sero-negative	-	+	RTX 2009 2012 AZA HCQ Pred	7.6
76	F	34	Af	5	dsDNA, ANA, ENA, Sm, RNP, C1q	-	+	RTX 2010 2012	6.8
77	F	51	Cauc	21	dsDNA, ANA, aPL	-	+	HCQ, Pred	26
78	F	23	Oriental	1	dsDNA, ANA, ENA, Ro	-	+	HCQ, Pred	2.2
79	F	54	Asian	40	ANA, aPL	+	-	HCQ, Pred	17.3
80	F	57	Cauc	14	dsDNA, ANA	+	+	HCQ, Pred	7.3
81	F	36	Cauc	14	ANA, ENA, Ro, Sm, RNP	-	+	RTX 2012 MMF HCQ Pred	2
86	F	60	Cauc	12	ANA, ENA, Ro, La	-	+	RTX 2012 MTX HCQ Pred	1.5
92	F	17	Af	10	dsDNA, ANA, ENA, Ro, Sm, RNP	+	+	RTX 2010 2012 Pred	6
95	F	32	Af	5	dsDNA, ANA, ENA, Ro, Sm, RNP, aPL	+	+	RTX 2012 MMF HCQ Pred	2.4
104	F	40	Cauc	20	dsDNA, ANA	+	+	RTX 2009 2012 AZA HCQ Pred	9.62
108	F	26	Af	7	dsDNA, ANA, ENA	+	+	RTX 2012 MMF HCQ Pred	26.3
8	F	55	Cauc	Unknown	sero-negative	-	+	Statins	0.26
118	F	62	Oriental	13	ANA, Ro	+	+	MMF, lisinopril, SIM,	22.15
119	F	47	AfC	13	dsDNA, ANA, Sm, RNP	+	+	Pred, AZA, LOS, RAN, Plaqenil	22.07
120	F	38	Cauc	16	sero-negative	+	+	AZA, Pred, warfarin, adalat, HCQ,	22.17
121	F	41	Cauc	7	dsDNA, ANA, Ro, Sm, RNP	+	-	CAN, HCQ, MTX, NTP, TRA, OMP,	10.68
122	F	37	Asian	12	ANA, Ro	+	+	unknown	21.84

124	F	25	Asian	13	sero-negative	+	+	Pred, myfortic,warfarin, VAL, ranitidine, HCQ,	40.7
125	F	24	Oriental	5	dsDNA, ANA, Ro, Sm, RNP	+	+	Pred., MTX, HCQ, MHC	22.49
126	F	46	AfC	21	ANA, RNP	+	+	Pred., aza, HCQ	0.78
127	F	41	AfC	18	dsDNA, ANA, La	-	+	Pred., MMF	1.22
128	F	56	AfC	3	dsDNA, Ro, Sm, RNP	+	-	Pred, HCQ, ramipril, MMF, alendronic cid	1.71
129	F	39	Asian	30	dsDNA, ANA	+	-	Pred, AZA, HCQ	3.12

Table S2. Demographic and clinical details of the GPA patients enrolled in this study

GPA

**Study #**	**M/F**	**Age**	**Ethnicity**	**BVAS on sample date**	**+ Auto-Ab status**	**Nephritis**	**Arthritis**	**Medication**
1	M	50	Cauc	19	cANCA	-	-	Pred
2	F	38	Cauc	21	cANCA	-	-	Pred MTX
3	M	49	Cauc	18	cANCA	+	-	Pred AZA
4	M	39	Cauc	26	cANCA	-	-	Pred MMF
5	M	56	Cauc	7	cANCA	-	-	Pred
6	F	67	Asian	18	cANCA	-	-	Pred
7	F	57	Cauc	15	cANCA	-	-	Pred MTX
9	M	27	Cauc	12	cANCA	-	-	Pred AZA
10	M	55	Cauc	18	cANCA	-	-	Pred AZA
11	F	45	Cauc	21	cANCA	-	-	Pred MMF
12	M	64	Cauc	30	cANCA	-	-	Pred MTX
13	M	41	Cauc	15	cANCA	-	-	Pred MT
14	M	27	Cauc	18	cANCA	+	-	Pred CYC IV
15	F	65	Cauc	4	cANCA	+	-	Pred MTX
16	F	65	Cauc	0	cANCA	+	-	Pred MMF
17	F	62	Cauc	6	cANCA	-	-	Pred AZA
18	M	65	Cauc	12	cANCA	-	-	Pred MTX
19	M	68	Cauc	0	cANCA	-	-	Pred MTX
21	M	65	Cauc	0	cANCA	-	-	Pred MTX
22	F	71	Cauc	6	cANCA	-	-	Pred MTX
23	F	58	Cauc	0	cANCA	-	-	Pred MTX
24	M	41	Cauc	5	cANCA	+	-	Pred MTX
25	M	64	Cauc	30	cANCA	-	-	Pred MTX

Table S3

Primer sequences

	Primer name	Primer sequence 5' to 3'
First round single cell PCR	VK1	CATCCAGWTGACCCAGTCTCC
	VK2	GATATTGTGATGACCCAGWCT
	VK3	GACRCAGTCTCCAGCCACCCTG
	VK4	GACATCGTGATGACCCAGTCT
	VK5	GAAACGACACTCACGCAGTCT
	VK6	GAAATTGTGCTGACTCAGTCT
	CK1N	TTAACACTCTCCCCTGTTGAAGC
	LVL1	GGTCCTGGGCCCAGTCTGTGCTG
	LVL2	GGTCCTGGGCCCAGTCTGCCCTG
	LVL3	GCTCTGTGACCTCCTATGAGCTG
	LVL4/5	GGTCTCTCTCSCAGCYTGTGCTG
	LVL6	GTTCTTGGGCCAATTTTATGGTG
	LVL7	GTTCTTGGGCCAATTTTATGCTG
	LVL8	GAGTGGATTCTCAGACTGTGGTG
	IGLC external	CCTTCCAGGCCACTGTCAC
Second round single cell PCR	VK1	CATCCAGWTGACCCAGTCTCC
	VK2	GATATTGTGATGACCCAGWCT
	VK3	GACRCAGTCTCCAGCCACCCTG
	VK4	GACATCGTGATGACCCAGTCT
	VK5	GAAACGACACTCACGCAGTCT
	VK6	GAAATTGTGCTGACTCAGTCT
	IGKC2	CTGTACTTTGGCCTCTCTGGG
	LVL1	GGTCCTGGGCCCAGTCTGTGCTG
	LVL2	GGTCCTGGGCCCAGTCTGCCCTG
	LVL3	GCTCTGTGACCTCCTATGAGCTG
	LVL4/5	GGTCTCTCTCSCAGCYTGTGCTG
	LVL6	GTTCTTGGGCCAATTTTATGGTG
	LVL7	GTTCTTGGGCCAATTTTATGCTG
	LVL8	GAGTGGATTCTCAGACTGTGGTG
	IGLC2	GCCACTGTCACRGCTCCCGGGC
First round intron / KDE	5’ JkCk intron E	CCGCGCTCTTGGGGCAGCCGCC
	3’ KDE E	CAGACAGGTCCTCAGAGGTC
Second round intron / KDE	JkCk intron I	GCCGCTATGCCGTGGCCACCC
	KDE I	CAGGTTGTCCTAGGGAGCAGGG

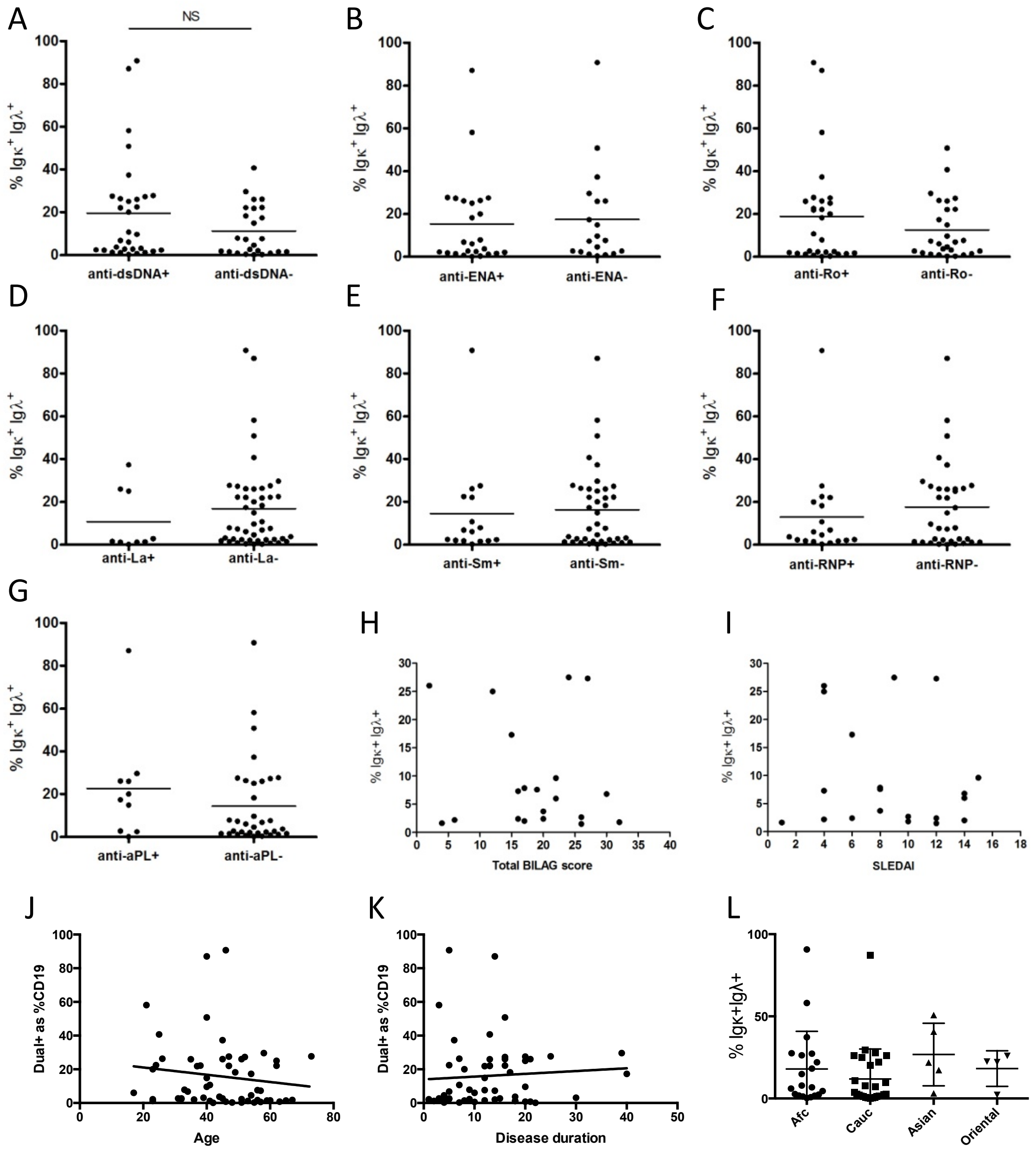

Figure S1. Dual kappa and lambda light chain expression by SLE patients positive and negative for the production of SLE-associated autoantibodies. (A) anti-dsDNA antibodies (B) anti-ENA antibodies (C) an#-Ro antibodies (D) anti-La antibodies (E) anti-Sm antigen antibodies (F) anti-RNP antibodies (G) anti-aPL antibodies. No association between dual light chain expression and (H) BILAG scores and (I) SLEDAI evaluation (J) age (K) Disease duration and (L) Disease activity.

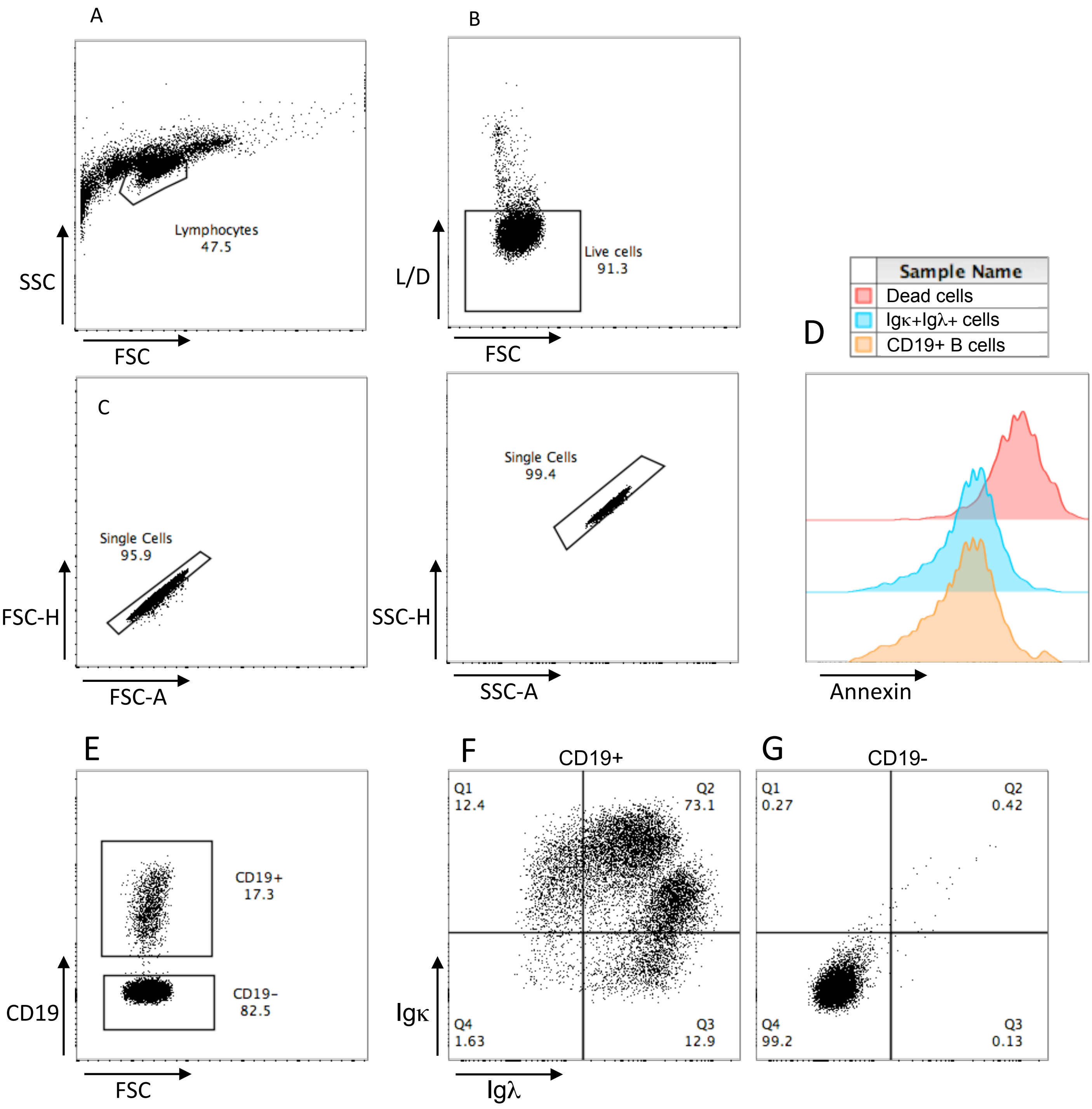

Figure S2. Examples of gating to identify A: lymphocytes B: Live cells by exclusion of live/ dead marker (L/D) C: to exclude doublets D: To confirm that cells expressin both Igκ and Igλ were annexin negative E: CD19+ B cells E: expression of Igκ and Igλ by B cells and F: cells other than B cells did not have assocaited surface light chains.
