# Supplementary material for: Immunoglobulin light chain allelic inclusion in systemic lupus erythematosus
Source: Eur J Immunol. 2015 Jun 24;45(8):2409–19. doi: 10.1002/eji.201545599 (PMC5102633; doi:10.1002/eji.201545599)
Supplement: Supplementary file 2 — Immunoglobulin light chain allelic inclusion in systemic lupus erythematosus [file EJI-45-2409-s002.pdf]

European Journal of Immunology

Supporting Information

for

DOI 10.1002/eji.201545599

Louise D. Fraser, Yuan Zhao, Pamela M. K. Lutalo, David P. D'Cruz, John Cason, Joselli S. Silva, Deborah K. Dunn-Walters, Saba Nayar, Andrew P. Cope and Jo Spencer

Immunoglobulin light chain allelic inclusion in systemic lupus erythematosus

© 2015 WILEY-VCH Verlag GmbH & Co. KGaA, Weinheim

European Journal of Immunology

Immunoglobulin light chain allelic inclusion in systemic lupus erythematosus.

Fraser, Louise; Zhao, Yuan; Lutalo, Pamela; D'Cruz, David; Cason, John; Silva, Joselli; Dunn-Walters, Deborah; Nayar, Saba; Cope, Andrew; Spencer, Jo

Correspondence: Dr. Spencer, Jo , New Guy's House, St Thomas' St, London, London Bridge, United Kingdom, SE1 9RT, Tel:44 207 188 1614, Fax: 44 207 188 3385

Review Timeline:	Submission date:	20-Feb-2015
	First Editorial decision:	17-Mar-2015
	Revision/s received:	23-Apr-2015
	Second Editorial decision:	08-May-2015
	Accepted:	29-May-2015

Handling Executive Committee member: Prof. Iain McInnes

Please note that the correspondence below does not include the standard editorial instructions regarding preparation and submission of revised manuscripts, only the scientific revisions requested and addressed.

First Editorial Decision - 17-Mar-2015

Dear Dr. Spencer,

Manuscript ID eji.201545599 entitled "Immunoglobulin light chain allelic inclusion in systemic lupus erythematosus." which you submitted to the European Journal of Immunology has been reviewed. The comments of the referees are included at the bottom of this letter.

A revised version of your manuscript that takes into account the comments of the referees will be reconsidered for publication.

You should also pay close attention to the editorial comments included below. **In particular, please edit your figure legends to follow Journal standards as outlined in the editorial comments. Failure to do this will result in delays in the re-review process.**

Please note that submitting a revision of your manuscript does not guarantee eventual acceptance, and that your revision will be re-reviewed by the referees before a decision is rendered.

If the revision of the paper is expected to take more than three months, please inform the editorial office. Revisions taking longer than six months may be assessed by new referees to ensure the relevance and timeliness of the data.

Once again, thank you for submitting your manuscript to European Journal of Immunology and we look forward to receiving your revision.

Yours sincerely,
Katharina Schmidt

On behalf of
Prof. Iain McInnes

Dr. Katharina Schmidt
Editorial Office
European Journal of Immunology
e-mail: ejied@wiley.com
www.eji-journal.eu

****

Reviewer: 1

Comments to the Author
The manuscript by Fraser and colleagues describes the expression of dual Ig light chains in patients with SLE. The paper is generally well written and interesting. The authors link expression of dual light chains to lower activity of the kappa deleting element; however, there are no mechanistic experiments performed to functionally connect reduced activity of KDE to dual light chain expression. Perhaps more importantly, I am not certain whether the manuscript offers any novelty to the field. In particular, the paper is entirely descriptive in nature, with no functional link made between dual IgL isotype expression and disease acquisition, maintenance, or progression. Without these mechanistic experiments, the paper may be better suited for another journal.

European Journal of Immunology

A few additional minor points:

The authors have a habit of using the phrase "in health". This does not make sense as written. All instances of this phrase should be revised to say, "in healthy donors", or "in healthy individuals", or "in healthy B cells".

Figure 2 adds very little to the story. Please move this to the supplementary materials.

Page 7 of 35: please include the PCR results for Igk+ and Iglambda+ in SLE patients and healthy donors as figure 4D.

Page 9: Typically, numbers less than 10 are spelled out ("...B cells from 2 healthy controls and 2 SLE patients...") while numbers greater than or equal to 10 are indicated by their numerals. Please correct these two instances.

Page 9: "...and to quantify N nucleotide additions". Please remove the "N".

Page 9: "flaning" should be "flanking".

Figure 1 and all FACS plots: My preference is to show dot plots, which are easier for me to visualize and interpret. Consider changing to dot plots.

Reviewer: 2

Comments to the Author
Rearrangement of the kappa deleting element (KDE) prevents double expression of Îº and Î» L-chains. Interestingly, patients with SLE frequently have B cells, which express both L chains. A similar finding was described previously in Lupus-prone mice and preliminary data were published for a few patients with SLE. This paper is a molecular analysis of allelic exclusion and shows for single B cells an inverse correlation between KDE rearrangement to the iRRS element and the presence of dual light chain expression. In addition, it analyzes the effect of IgÎº and IgÎ» dual expression on SLE development, however in its conclusions, the paper and in particular the discussion are rather vague.
The paper shows that allelic inclusion was not associated with autoantibody titers, or disease activity. However, in the relatively small cohort of 130 SLE patients, two individuals were found where practically all of the B cells showed dual IgÎº and IgÎ» expression (figs 1 and 2). Further information on these two patients is missing and this amazing finding is not even mentioned in the text. I would think, that these findings give

European Journal of Immunology

strong evidence, that deficiency in KDE rearrangement predisposes to the development of SLE. In this respect, it would be important to test B cells from patients with diseases like rheumatoid arthritis, SjÃ¶grenÂ´s syndrome or scleroderma to determine whether dual IgÎº and IgÎ» expression is a general phenomenon in autoimmune diseases.

â€¢ The percentage of SLE patients with dual IgÎº and IgÎ» expressing B cells should be calculated and the average percentage of bi-specific B cells in those patients determined

Sorting of single dual IgÎº and IgÎ» expressing plasma blasts will increase the efficiency of both IgÎº+ and IgÎ»+ PCR amplicons

Fig. 3 abbreviation â€œRep #1 and Rep #2â€• , please explain

Fig 4A what is shown in lower panels, what is the meaning of FMO

Please correct: â€œthe sequences flaning them (page 9)â€•

Reviewer: 3

Comments to the Author

In their report "Immunoglobulin light chain allelic inclusion in systemic lupus erythematosus" Fraser et al. present a compelling and comprehensive overview of the IgK+/IgL+ dual expression phenomenon in healthy donor control subjects and clinical patients diagnosed with SLE. They report that SLE patients have markedly higher levels of light chain allelic inclusion, and that allelic inclusion levels were even higher in SLE patients with lupus nephritis; however, dual light chain expression did not correlate with overall disease activity score. They also found that allelic inclusion was observed across the majority of B-cell subsets, and performed several control experiments (e.g. removal of dsDNA and surface-bound Ig, cell fixation and staining, etc.) to verify their FACS allelic inclusion data. Finally they observed that kappa-deleting element (KDE) recombinations with the intronic recombination signal sequence (iRSS) were less prevalent in SLE patients, which correlated well with the prevalence of allelic inclusion within donors.

The research presented here is well-written, well-described, adequately controlled, and balanced in its interpretation of the results. The results reported are novel and have the potential to dramatically alter our understanding of SLE once we gain a greater understanding of why allelic inclusion occurs at a greater frequency in SLE patients, and/or if allelic inclusion can be demonstrated as a contributing factor or cause of SLE disease in follow-up studies. The prospect of mechanistic follow-up studies which build upon this report is considerable.

European Journal of Immunology

Major comments:

All additional data/amplifications requested (see below) should already be available to the authors.

Page 6: The authors declare â€œThis was not a feature of dying cells and was not a feature of PBMC other than the B cell subsets described (data not shown).• Although I appreciate that this is negative• data, I feel it is mandatory that this be documented as supplemental data to accompany Figure 3. It would behoove the authors to demonstrate a representative FACS diagram which shows the complete gating series of PBMCs (e.g., FSC v. SSC, viability staining, CD19- v. CD19+ cells in regards to light chain staining).

Page 7: Please report efficiency of sequence recovery for IgK+, IgL+, and IgK+L+ single-cell RT-PCR analysis groups (both Healthy Controls and SLE). For example, of the 394 single SLE B cells analyzed, how many yielded IgK sequences, how many yielded IgL sequences, and how many yielded both? And the same for healthy donors. Some speculation regarding the disparate efficiencies are mentioned in the discussion section, and reporting all these values will be helpful for understanding the present work and important to provide a basis for further studies.

Supporting Table II - Could additional columns be added to Supporting Table II that report the data points for Fig 1e? Being able to track the % included IgK/IgL cell fraction by individual patient may be useful for future studies and reveal age-related or genetic-related patterns. Also a quick regression analysis of those data by the various patient characteristics may be helpful in the present report. If the authors identify any correlates of % allelic inclusion according to age, ethnicity, disease duration, or auto-Ab status reported in Supporting Table II, that might be extremely helpful for the field and a very interesting result.

Minor comments:

Page 10 lines 7-8 - "Light chain isotypic EXCLUSION was more common in B cells from SLE patients with more severe disease (as manifest by lupus nephritis)" I think I see K-L allelic INCLUSION to be more common in patients with nephritis (Fig 1e). I believe this is a typo, please check.

Figure 6b - Please clarify with units on n values in the figure (e.g. n = x donors, n = y cells) for clarity. I understand it after spending time with the manuscript, but seeing two different n values on the same figure was very confusing to me at first pass.

European Journal of Immunology

<u>**First Revision – authors' response – 23-Apr-2015**</u>

Responses to reviewers' comments

We are grateful to the reviewers for their very helpful comments. Our responses and changes to the manuscript are described below. The reviewers' comments are in italic with our responses in plain type below. Changes are marked in in the manuscript in red type.

Reviewer: 1

Comments to the Author

The manuscript by Fraser and colleagues describes the expression of dual Ig light chains in patients with SLE. The paper is generally well written and interesting. The authors link expression of dual light chains to lower activity of the kappa deleting element; however, there are no mechanistic experiments performed to functionally connect reduced activity of KDE to dual light chain expression. Perhaps more importantly, I am not certain whether the manuscript offers any novelty to the field. In particular, the paper is entirely descriptive in nature, with no functional link made between dual IgL isotype expression and disease acquisition, maintenance, or progression. Without these mechanistic experiments, the paper may be better suited for another journal.

We are pleased that this reviewer finds our manuscript interesting. The reviewer is concerned however about the lack of mechanistic experiments. Whilst we understand this concern, this study reports an original and clinically relevant finding relating to lymphocytes in human SLE that is supported by previous studies of animal models of lupus. We propose inefficiency of rearrangement of the KDE as a mechanism behind allelic inclusion in SLE and present the data to support this. We consider that our original observations together with the proposed underlying molecular basis and clinical relevance constitute an important advance that is suitable for publication in the *European Journal of Immunology*.

A few additional minor points:

The authors have a habit of using the phrase "in health". This does not make sense as written. All instances of this phrase should be revised to say, "in healthy donors", or "in healthy individuals", or "in healthy B cells".

Apologies, this has been changed throughout the manuscript.

Figure 2 adds very little to the story. Please move this to the supplementary materials.

This has been removed from the manuscript and is now Figure S1 and includes more comparisons.

Page 7 of 35: please include the PCR results for Igk+ and Iglambda+ in SLE patients and healthy donors as figure 4D.

Data is now included in Figure 1 F and G and is referred to in the text on page

European Journal of Immunology

Page 9: Typically, numbers less than 10 are spelled out ("...B cells from 2 healthy controls and 2 SLE patients...") while numbers greater than or equal to 10 are indicated by their numerals. Please correct these two instances.

Page 9: "...and to quantify N nucleotide additions". Please remove the "N".

This has been done

Page 9: "flaning" should be "flanking".

Figure 1 and all FACS plots: My preference is to show dot plots, which are easier for me to visualize and interpret. Consider changing to dot plots.

We experimented with presenting data in many different ways and revisited this in response to this comment. We still feel that the contour plots identify best where there appears to be a separate population of cells and where populations merge. However, in response to this comment we used dot plots for a new component to Figure 2 so that the data can be viewed both ways in the manuscript.

Reviewer: 2

Comments to the Author

Rearrangement of the kappa deleting element (KDE) prevents double expression of κ and λ L-chains. Interestingly, patients with SLE frequently have B cells, which express both L chains. A similar finding was described previously in Lupus-prone mice and preliminary data were published for a few patients with SLE. This paper is a molecular analysis of allelic exclusion and shows for single B cells an inverse correlation between KDE rearrangement to the iRRS element and the presence of dual light chain expression. In addition, it analyzes the effect of Igκ and Igλ dual expression on SLE development, however in its conclusions, the paper and in particular the discussion are rather vague.

We have edited the discussion throughout so that it is more incisive.

The paper shows that allelic inclusion was not associated with autoantibody titers, or disease activity. However, in the relatively small cohort of 130 SLE patients, two individuals were found where practically all of the B cells showed dual Igκ and Igλ expression (figs 1 and 2). Further information on these two patients is missing and this amazing finding is not even mentioned in the text. I would think, that these findings give strong evidence, that deficiency in KDE rearrangement predisposes to the development of SLE.

The individual patients have now been aligned with the data in Table S1 so that the relationship between patients and data is transparent. Features of the dual light chain positive populations have been provided in more detail in the text on page 5.

In this respect, it would be important to test B cells from patients with diseases like rheumatoid arthritis, Sjögren´s syndrome or scleroderma to determine whether dual Igκ and Igλ expression is a general phenomenon in autoimmune diseases.

We had already studied an additional group of patients with autoimmune disease granulomatosis with polyangiitis (GPA). This data that resembles the data from healthy controls is now included in Figure 1E

European Journal of Immunology

and a new supplementary table S2 with patient details is included.

- *The percentage of SLE patients with dual Igκ and Igλ expressing B cells should be calculated and the average percentage of bi-specific B cells in those patients determined*

This is now included in the results on Page 5.

- *Sorting of single dual Igκ and Igλ expressing plasma blasts will increase the efficiency of both Igκ+ and Igλ+ PCR amplicons*

This has been clarified in the manuscript. Unfortunately the PCR was inefficient even in sorted Igκ+, Igλ+ cells.

- *Fig. 3 abbreviation "Rep #1 and Rep #2", please explain*

These are 2 example replicates and this has been clarified in the figure

- *Fig 4A what is shown in lower panels, what is the meaning of FMO*

This is fluorescence minus one control and this has been clarified.

- *Please correct: "the sequences flaning them (page 9)"*

Apologies, this has been corrected.

Reviewer: 3

Comments to the Author

In their report "Immunoglobulin light chain allelic inclusion in systemic lupus erythematosus" Fraser et al. present a compelling and comprehensive overview of the IgK+/IgL+ dual expression phenomenon in healthy donor control subjects and clinical patients diagnosed with SLE. They report that SLE patients have markedly higher levels of light chain allelic inclusion, and that allelic inclusion levels were even higher in SLE patients with lupus nephritis; however, dual light chain expression did not correlate with overall disease activity score. They also found that allelic inclusion was observed across the majority of B-cell subsets, and performed several control experiments (e.g. removal of dsDNA and surface-bound Ig, cell fixation and staining, etc.) to verify their FACS allelic inclusion data. Finally they observed that kappa-deleting element (KDE) recombinations with the intronic recombination signal sequence (iRSS) were less prevalent in SLE patients, which correlated well with the prevalence of allelic inclusion within donors.

The research presented here is well-written, well-described, adequately controlled, and balanced in its interpretation of the results. The results reported are novel and have the potential to dramatically alter our understanding of SLE once we gain a greater understanding of why allelic inclusion occurs at a greater frequency in SLE patients, and/or if allelic inclusion can be demonstrated as a contributing factor or cause of SLE disease in follow-up studies. The prospect of mechanistic follow-up studies which build upon this report is considerable.

Major comments:

European Journal of Immunology

All additional data/amplifications requested (see below) should already be available to the authors.

Page 6: The authors declare “This was not a feature of dying cells and was not a feature of PBMC other than the B cell subsets described (data not shown).” Although I appreciate that this is “negative” data, I feel it is mandatory that this be documented as supplemental data to accompany Figure 3. It would behoove the authors to demonstrate a representative FACS diagram which shows the complete gating series of PBMCs (e.g., FSC v. SSC, viability staining, CD19- v. CD19+ cells in regards to light chain staining).

This additional information and the gating strategy are included in a new Figure S2, and new additions to Figure 2 (parts A and B).

Page 7: Please report efficiency of sequence recovery for IgK+, IgL+, and IgK+L+ single-cell RT-PCR analysis groups (both Healthy Controls and SLE). For example, of the 394 single SLE B cells analyzed, how many yielded IgK sequences, how many yielded IgL sequences, and how many yielded both? And the same for healthy donors. Some speculation regarding the disparate efficiencies are mentioned in the discussion section, and reporting all these values will be helpful for understanding the present work and important to provide a basis for further studies.

This is now on page 5.

Supporting Table II - Could additional columns be added to Supporting Table II that report the data points for Fig 1e? Being able to track the % included IgK/IgL cell fraction by individual patient may be useful for future studies and reveal age-related or genetic-related patterns.

A new column in the supporting table has been included.

Also a quick regression analysis of those data by the various patient characteristics may be helpful in the present report. If the authors identify any correlates of % allelic inclusion according to age, ethnicity, disease duration, or auto-Ab status reported in Supporting Table II, that might be extremely helpful for the field and a very interesting result.

Thank you for this suggestion. Nothing significant was found, but this has now been included in Figure S1 that is a revised version of old Figure 2.

Minor comments:

Page 10 lines 7-8 - "Light chain isotypic EXCLUSION was more common in B cells from SLE patients with more severe disease (as manifest by lupus nephritis)" I think I see K-L allelic INCLUSION to be more common in patients with nephritis (Fig 1e). I believe this is a typo, please check.

Apologies, this has been corrected.

Figure 6b - Please clarify with units on n values in the figure (e.g. n = x donors, n = y cells) for clarity. I understand it after spending time with the manuscript, but seeing two different n values on the same figure was very confusing to me at first pass.

Apologies, this has been corrected.

Second Editorial Decision - 08-May-2015

Dear Dr. Spencer,

It is a pleasure to provisionally accept your manuscript entitled "Immunoglobulin light chain allelic inclusion in systemic lupus erythematosus." for publication in the European Journal of Immunology. For final acceptance, please follow the instructions below and return the requested items as soon as possible as we cannot process your manuscript further until all items listed below are dealt with.

Please note that EJI articles are now published online a few days after final acceptance (see Accepted Articles: http://onlinelibrary.wiley.com/journal/10.1002/(ISSN)1521-4141/accepted). The files used for the Accepted Articles are the final files and information supplied by you in Manuscript Central. You should therefore check that all the information (including author names) is correct as changes will NOT be permitted until the proofs stage.

We look forward to hearing from you and thank you for submitting your manuscript to the European Journal of Immunology.

Yours sincerely,
Katharina Schmidt

on behalf of
Prof. Iain McInnes

Dr. Katharina Schmidt
Editorial Office
European Journal of Immunology
e-mail: ejied@wiley.com
www.eji-journal.eu
